# The Effects of Spatially Heterogeneous Prey Distributions on Detection Patterns in Foraging Seabirds

**DOI:** 10.1371/journal.pone.0034317

**Published:** 2012-04-13

**Authors:** Octavio Miramontes, Denis Boyer, Frederic Bartumeus

**Affiliations:** 1 Departamento de Sistemas Complejos, Instituto de Física, Universidad Nacional Autónoma de México, Mexico City, México; 2 C3, Centrso de Ciencias de la Complejidad, Universidad Nacional Autónoma de México, Mexico City, México; 3 Departamento de Física, Universidade Federal do Paraná, Curitiba, Brasil; 4 Centre d’Estudis Avançats de Blanes, Blanes, Girona, España; University of Utah, United States of America

## Abstract

Many attempts to relate animal foraging patterns to landscape heterogeneity are focused on the analysis of foragers movements. Resource detection patterns in space and time are not commonly studied, yet they are tightly coupled to landscape properties and add relevant information on foraging behavior. By exploring simple foraging models in unpredictable environments we show that the distribution of intervals between detected prey (detection statistics) is mostly determined by the spatial structure of the prey field and essentially distinct from predator displacement statistics. Detections are expected to be Poissonian in uniform random environments for markedly different foraging movements (*e.g.* Lévy and ballistic). This prediction is supported by data on the time intervals between diving events on short-range foraging seabirds such as the thick-billed murre (*Uria lomvia*). However, Poissonian detection statistics is not observed in long-range seabirds such as the wandering albatross (*Diomedea exulans*) due to the fractal nature of the prey field, covering a wide range of spatial scales. For this scenario, models of fractal prey fields induce non-Poissonian patterns of detection in good agreement with two albatross data sets. We find that the specific shape of the distribution of time intervals between prey detection is mainly driven by meso and submeso-scale landscape structures and depends little on the forager strategy or behavioral responses.

## Introduction

A number of seabird species search and catch prey in ranges from hundreds to thousands of kilometers away from their nesting sites [1]–[8]. The changing nature of marine environments makes seabird prey distributions highly dynamic and unpredictable over large spatial scales, ultimately impacting on seabirds capture efficiency [9], [10]. In this scenario, seabird populations are under constant survival pressure, a situation worsened by climate change, that significantly perturb prey availability and the ecology of predator-prey systems [11], [12]. A well known example is the impact of El Niño-ENSO oscillations in the Pacific Ocean [13] on sardine population fluctuations off South Africa coast [8]. Studies of how seabirds detect and catch prey in the open ocean are also very important to assess the health of fish stocks [14]–[18], particularly for declining species that are commercially valuable [19], [20]. The availability of telemetry and satellite tracking technologies [21]–[25] accounts for recent progress in the understanding of habitat use and foraging behavior of long-range oceanic birds [26], [27]. Yet, this new empirical knowledge has been seldom followed up by theoretical studies providing general and more formal rationale for the observed foraging patterns. Motivated by this, and inspired by the long-range foraging patterns of albatrosses, here we explore how landscape-properties (*i.e.* large-scale prey spatial distributions) affect prey detection patterns in seabirds.

Foraging models (see, *e.g.* [28]–[30]) often examine the average distance (or time) travelled between successive prey detections, a key quantity that is inversely proportional to the foraging efficiency. Much less attention has been paid to the entire distribution of distances/times between detected prey (but see [30]), herein referred to as detection statistics. This latter quantity has been sometimes directly measured, in particular for wandering albatrosses (*Diomedea exulans*) [10], [31]. It is worth noting that detection patterns in unpredictable environments are -*a priori*- not closely related to displacement patterns. Displacements, *i.e.*, a set of positions defining a trajectory, reflect internal states and complex behavioral responses to resource distributions [32]–[37]. Detections, in turn, are localized events resulting from the explicit or physical interaction of the forager with the prey field and/or targeted landscape features.

For the past decade, a wide debate has focused on animal movement models with power-law move length distributions (Lévy walks) and on their possible interpretation as optimal search strategies of randomly distributed prey [28], [38]–[41]. The movement patterns of many foragers, for instance, marine predators [37], plankton [42], spider monkeys [43] or jackals [44] display a wide range of spatial scales that cannot be accounted for by Poisson statistics. Wandering albatrosses were actually one of the first biological examples where evidence for Lévy displacements was reported [24], [28]. Flaws found later in the analysis questioned these findings and data of higher resolution were neither fitted by a Lévy law nor a Poisson law, but by a truncated modified power-law function [45]. This set of studies has attempted to draw conclusions on the search strategies of albatrosses not from direct position tracking, but based on flight duration data, which were assumed to be indicative of detection times between prey [24], [28], [45]. Here we provide further evidence showing that these data are actually related to detections, but also show that they do not carry information on movements and, therefore, on the nature of the search patterns leading to these detections.

We more generally examine the effects of the prey field spatial structure and of foraging rules on detection patterns. For prey uniformly distributed in space, detection patterns are trivially exponential if displacements are ballistic or self-avoiding but the outcome is less obvious for other types of movement. We find that Lévy movement models [28] also lead to exponential prey detection patterns in Poissonian environments, which illustrates the markedly different nature of detection and movement statistics. These predictions can explain the diving patterns of short-range foraging seabirds, such as the thick-billed murre [46], whose dives are exponentially distributed on time.

Prey in the ocean are not uniformly distributed at large scales, however [47]–[50]. Detection patterns in complex media have been little studied and mostly in non-biological contexts [51]. We show that the non-Poissonian albatross data of [10] and [45] can be explained by models of a forager flying over a fractal prey landscape with parameter values consistent with observed resource distributions in the ocean. We use two models generating fractal landscapes of different nature and relate the fluctuations in the predator detection times (or distances) to the prey density heterogeneities. As in the uniform case, detection patterns in a given environment are found robust with respect to a variety of foraging rules, where the predator may or may not switch between different behaviors depending on prey detection.

## Analysis

### Foraging Seabirds: Movement vs. Detection Statistics

In this study we re-analyze data from thick-billed murres and wandering albatrosses, two seabird species with markedly different behaviors. Thick-billed murres forage over small spatial scales in short foraging trips (representing less than 1h of flight in total) within a few kilometers of their colony [46]. They feed on benthic or pelagic fish in zones where prey occur in patch and are relatively predictable. These animals show a high degree of site fidelity. Murres perform above-water and underwater searching, although the latter has a much shorter mean duration [46]. In ref. [46], flight durations (*t*) of thick-billed murres between consecutive dives were measured with time-depth-temperature recorders.

On the other hand, telemetry data reveal that some albatrosses species, especially wandering albatrosses, perform exploratory trips of thousands of kilometers involving commuting and looping typical of central-place foraging [10], [52]. This large scale behavior is interspersed with hierarchically nested area-restricted search induced by the recognition of water masses such as the shelf edge, sea-mounts or frontal zones. Prey are likely to be scattered within these mesoscale physical structures that represent higher profitability areas that need to be prospected, involving successive landings and take-offs [52]. Heart-rate recorder signals in wandering albatrosses show that landings and take-offs represent a high energy expenditure for these large birds, who practically consume as much energy flying with a favorable tail or side wind as when sitting on the water or resting on the nest [53]. From an optimality standpoint landings should be considered informed behavioral responses, mostly associated to prey detection or exclusive seascape features, but not strictly related to successful prey captures. In [10], [54] it was observed that birds need about two landings on average before capturing prey (measured from stomach temperature sensors data). In particular, two capture modes have been identified in wandering albatrosses: “foraging in flight”, where the prey is captured within a few seconds after landing, and “sit-and-wait”, where the bird is sitting on the water for more than 10 min before prey is caught [31], [52]. The sit-and-wait strategy appears to be a secondary tactic used for prey clustered in small patches, for which foraging in flight would require high turning and landing rates, or for prey capture at night [52]. Albatrosses also land in water to rest, probably selecting the resting areas as well. Herein the term “prey detection” will denote the detection of prey, prey cues, or targeted seascape areas (for prospection, potential prey captures, resting, etc.) that may induce landing or diving responses.

One of the wandering albatross data discussed in the following (Bird Island data [45]) were obtained with wet-dry sensors measuring flight durations (*t*) between successive take-offs and landings. The data was acquired in 2004 with a reading each 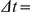
10s [45]. However this technology, which is similar to that of the murre data mentioned above, does not give information on trajectories and the animals were not equipped with a high resolution GPS device. The second wandering albatross data set re-analyzed here (Crozet Islands data [10]) consists in distances between captured prey measured using stomach temperature transmitters and position tracking systems.

Let us now consider, as an illustrative example, the search model of [28]. A forager with constant velocity *v* chooses randomly oriented, rectilinear displacements of lengths (*l*) drawn from a probability distribution function (PDF) 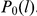 Prey is immobile and randomly, uniformly distributed on a plane in number density 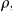 and the forager can detect a prey only when it is at a shorter distance than a perception radius *r*. A step is stopped if a prey is detected on the way or completed otherwise.

Viswanathan *et al.* [28] considered power-law distributions, 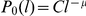 for 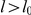 and zero otherwise, where 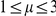. To test this move length distribution for wandering albatrosses, [28] and [45] compared the PDF of the flight durations *t* obtained from the wet-dry sensors to a power-law distribution. A similar comparison was performed with the flight duration data of the thick-billed murres [46]. In these studies, *t* was thus assumed to be indicative of a chosen move length, *l*. But, as we have argued, *t* represents the time elapsed between two detections, not a time spent traveling in straight line between two re-orientations. As *t* and *l* are different variables, they *a priori* obey different distributions. Therefore, comparing the model (or any other foraging model) with the three data sets described above requires to seek the PDF of the distance flown between two successive detected prey for that model, denoted as *L* here (if the bird velocity is constant, then *L*  =  *vt*). Equivalently, *L* is the sum of the step lengths travelled between prey. One may use the identity 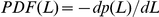, where 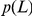 is the probability that a path of length *L* has not found a prey yet (or the fraction of flights of length 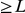).

## Results

### Prey Detections in Poissonian Landscapes

**Figure 1:**
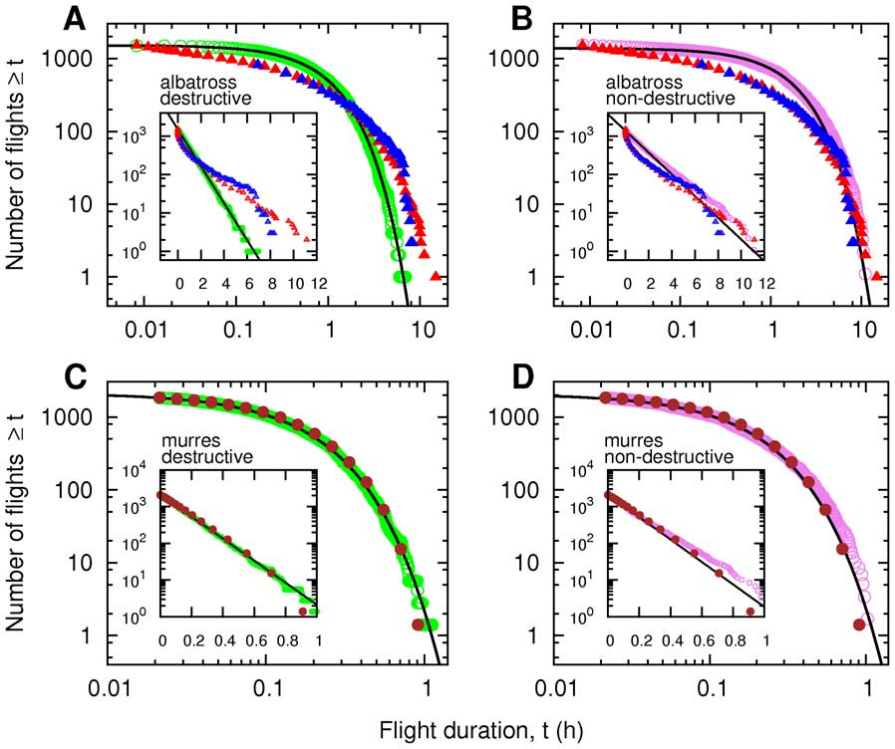
Detections in random uniform prey landscapes. (A) Albatross data and the model with destructive scenario. Green circles: accumulated distribution 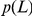 of flight lengths between successive detected prey of the model forager [28] with perception radius *r* = 0.001 following a Lévy process with *µ* = 1.5 (from 20 simulations of 75 captures each). The foraging ground is represented by a square of area unity and contained 5000 prey. Continuous line: exponential fit. Red triangles: 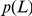 of the Bird Island albatross takeoff/landing data [45]. Blue triangles: 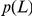 of the Crozet Islands albatross prey capture data [10], converted into flight durations assuming a constant flight velocity 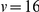
m/s. Inset: same curves represented in semi-log to better emphasize the non exponential nature of the observed albatross data versus the exponential form of the model forager detections. (B) Albatross data and model with non-destructive scenario. Violet circles: accumulated distribution 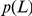 for the model forager performing a Lévy process with *µ* = 2. Continuous line: exponential fit. Prey number: 3000; *r* = 0.0003. In A) and B), the lengths in the model with foraging arena of area unity are converted in hours (*t*) by using 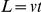 with the scaling factor *v* = 0.12. Inset: same curves represented in semi-log to better emphasize the non exponential nature of the observed albatross data versus the exponential form of the model forager detections. (C) Murre data and model with destructive scenario. Green circles: accumulated distribution 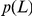 of flight lengths between prey of the model forager with perception radius *r* = 0.001 following a Lévy process with *µ* = 1.5 (from 20 simulations of 75 captures each). Continuous line: exponential fit. Brown dots are the murre flight durations from [46]. Inset: same data represented in semi-log in order to better emphasize the exponential nature of both the observed murres data and the model forager. (D) Murre data and model with non-destructive scenario. Violet circles: accumulated distribution 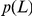 for a forager performing a Lévy process with *µ* = 2. Continuous line: exponential fit. Prey number: 3000; *r* = 0.0003. Inset: same curves represented in semi-log. Similar close-to-exponential detections are obtained in all simulations with 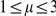, in both destructive and non-destructive scenarii.

We illustrate below that the distance flown between two successive detection events, i.e. *L*, is exponentially distributed in random and uniform prey fields, even if the choice distribution 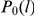 is not an exponential. In such landscapes, if detected prey disappear (destructive scenario), any foraging strategy producing paths that do not revisit the same location is optimal. Such non-oversampling paths can be ballistic (similar to a Lévy process with 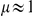), spirals, self-avoiding walks, etc. Any non-oversampling path of length *L* has a probability 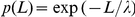 of not finding any prey, with 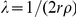 a characteristic distance, being *r* a detection radius and 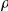 the prey density. If the forager follows a random Lévy search, its trajectory involves some degree of oversampling. We have obtained 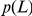 from numerical simulations for this model. In a destructive scenario, in which prey are depleted and not revisited, 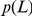 closely follows an exponential, not only for 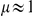 but also for walks with 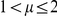 (Figure 1A and C). In the non-destructive scenario, prey can be revisited. If one chooses 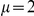 or any smaller value, one also observes exponential detection statistics in a very good approximation (Figure 1B and D). The distribution 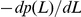 has the form 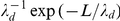, a shape which is not related to that of 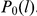 These results illustrate that exponential tails for prey detection statistics are an essential outcome of foraging models, including those generated from Lévy processes, when the landscape is Poissonian. However, the precise value of the characteristic length travelled between prey, 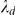 (which is related to the foraging efficiency), generally depends on the scenario and movement rules (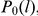
here). As we assume that movement is truncated by detections, 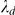 is finite.

The simple exponential form of 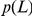 obtained for uniform prey fields describes well the murre data. The maximum likelihood estimate (MLE) of 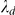 is 9.5 min and a log-likelihood ratio test of goodness-of-fit (G-test) was performed from 10^4^ independent Monte Carlo samplings, giving 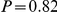 (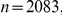 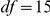). In contrast, the exponential does not describe the wandering albatross curves, see Figure 1 (G-test, Bird Island: 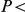 0.0001, *n* =  1507, 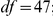 Crozet Islands: 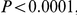 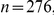 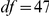). In this figure, distances in the Crozet I. data were converted into flight durations assuming a constant flight velocity 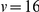
m/s [52], [55]. The resulting curve lies very close to the Bird Island data.

### Prey Detection in Large Scale Fractal Landscapes

In the case of Bird Island wandering albatrosses, Edwards *et al.* accurately fitted the flight duration distribution by a shifted gamma function, which is asymptotically an exponential multiplied by an inverse power-law [45]. Similarly, Weimerskirch *et al.* found that the distribution of distances between captured prey by Crozet Islands wandering albatrosses did not follow a simple exponential, but approximately an inverse power-law [10] (see also [56]).

Such intermittent landing by albatrosses, often related to prey capture behavior, can be explained by fractal prey landscapes. As a matter of fact, wandering albatrosses forage over much larger spatial scales than murres and mainly feed on squid and pelagic fish [10]. This prey display several levels of spatial aggregation and schooling [47], [48] and have strong spatial overlap with plankton [49]. The large scale horizontal spatial distributions of plankton [37], [50], passive drifters [57], cephalopods [47] and pelagic fish [48], [58]–[60] are known to be self-similar (with fractal dimension 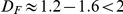) over a wide range of scales, typically from a lower characteristic scale 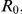 of tens of meters, to an upper scale 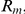 of 100−300 km [50], [58], [60]. At scales larger than 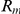 the prey field is seen as heterogeneous but space filling, that is, with 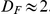 The mechanisms generating fractal horizontal distribution of marine species near the ocean surface are not well-known. Oceanic turbulence [50], [60] and predator-prey interactions [61] are two factors often invoked.

Based on these field observations, we consider below more realistic prey distribution models that generate fractals of different types.

#### (a) Truncated Lévy Dust model (LD)

It is commonly accepted that the assumption of randomly distributed prey in spatial ecological models is not entirely appropriate since there is a growing body of evidence showing that prey are more likely distributed in a patchy and aggregated fashion. This seems to be especially true for distributions of prey in marine environments as discussed above [48], [58]–[60]. Lévy dusts in finite domains are a convenient method to generate stochastic fractal point patterns and they have been applied to model oceanic prey fields [37], [50]. They have been less often used to model the movement of foragers profiting on these, however (but see [62]). Our first fractal foraging model therefore employs truncated Lévy dusts (LD) to generate fractal prey locations.

**Figure 2:**
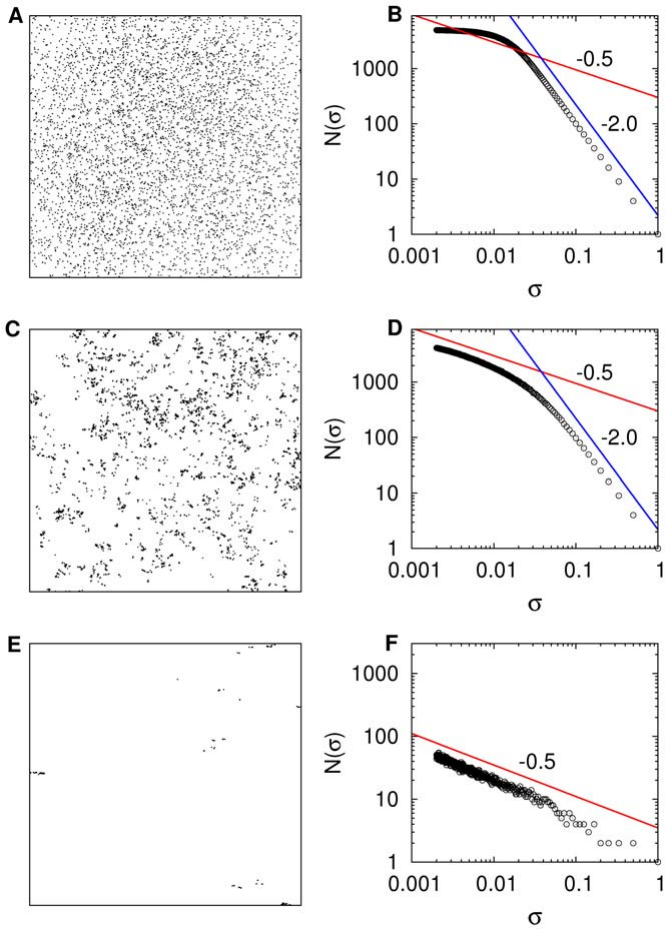
Three different theoretical patterns of spatial prey distribution in a unit box and their corresponding box-counting fractal dimension. 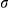
 represent the size of the boxes and 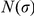 is the number of boxes of size 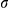 in the box-counting algorithm. In the three cases, 5000 prey are distributed accordingly to a Lévy dust with fractal dimension 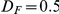 (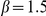). (A) If the minimal distance between prey is large the Lévy process producing the fractal pattern bounces many times on the walls and the overall process tends to be space-filling. In this particular case, the minimal distance between prey was 1/7 and the process bounced around 2500 times which is equivalent to the superposition of 2500 separated fractals in the same domain. (B) As expected in this case, the fractal dimension measured by box-counting does not show a scaling region with exponent 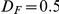 (red line) but approximates more the typical graph of a 2D random process with 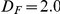 (blue line). (C) Pattern that corresponds to a prey distribution with a minimal distance of 1/700 between prey, leading to less than 150 bounces (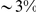 of the total prey number). (D) In this case a scaling region with 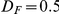 is visible, followed by a two-dimensional behavior at larger length scales. (E) A very clumped and aggregated fractal pattern of prey is obtained when the minimal distance between prey is set to 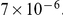 (F) In this case the fractal is nearly perfect with 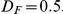

**Figure 3:**
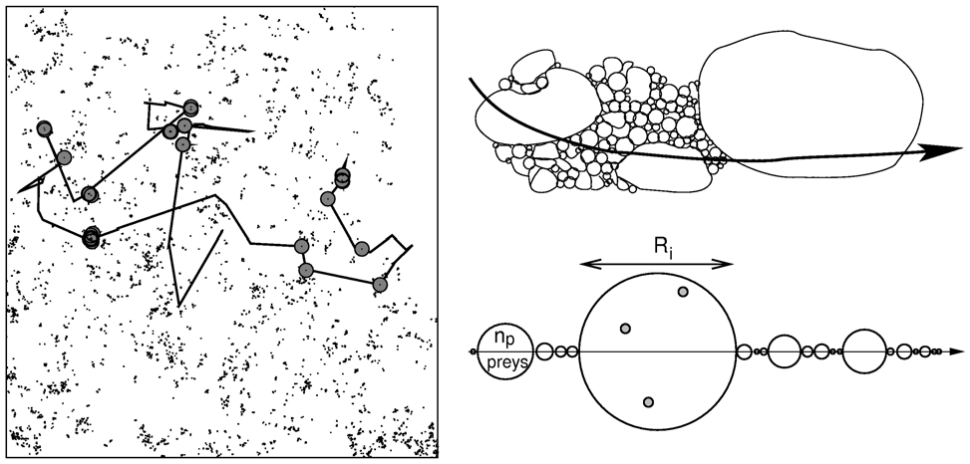
Left panel. Foraging arena composed of *N* = 5000 prey generated with a LD of exponent 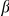 = 1.5 (fractal dimension 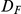 = 0.5). Solid line: trajectory of a ballistic forager (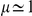) with detection radius *r* = 0.001. The larger grey dots indicate detection events (destructive scenario). Right panels: Fractal Local Density (FLD) model. Upper figure: The medium is composed of patches of heterogeneous sizes *R*, drawn from a PDF 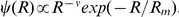 Within a patch, 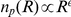 prey are randomly and uniformly distributed. Lower figure: linear representation of the forager/medium system, which is solved here.

LD are standard Lévy flights coming from the power-law distribution 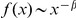 with 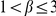 [63] and where only the turning points joining successive displacements *x* are considered as prey locations. This method generates point patterns with fractal dimension 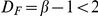 (Figure 2). The power-law distribution when finite (contained in a square domain-box of unit length) is truncated in the range 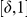 where 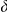 is interpreted as the minimum distance separating neighboring prey. On the other hand, the maximum distance separating consecutively located prey is the domain-box size, set to 1 for convenience. Between both limits (which define the self-similarity range of the fractal) the corresponding truncated probability distribution function is normalized to 1. The Lévy dust generator starts at the center of a square domain of unitary area and accommodates *N* successive prey (see Figure 2). When a new prey position is to be located outside of the domain, it is discarded and a new step is attempted (we call this a “border-bounce”). The fractal nature of the pattern may disappear if the number of bounces is too high. In order to prevent this, a tuning of 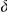 is applied to guarantee that the number of bounces is low, given a total number of prey. If the distance 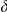 is large enough (but always 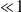) and if the total number of prey is also large, the pattern approximates a Poisson distribution because of too much bouncing. If the value of 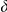 is too small, the prey field is limited to a very small region of the domain. An intermediate situation would produce a locally sparse fractal covering the whole domain. In our simulations, we took values of 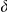 such that a bounce occurs in no more than 1.5% of the total number of prey. It is also necessary to keep in mind that the value of 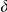 depends on the value of the scaling exponent 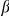 of the walker. The lower the exponent 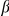 is, the lower the value of 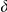 has to be in order to generate an undistorted fractal with few bounces (see Figure 2). We will discuss in the following section the detection dynamics of a forager moving on a fractal prey field generated by this process (see Figure 3, left).

#### (b) Fractal Local Density (FLD) model

We next propose an alternate and original model that builds stochastic fractals where, in contrast with Lévy dusts or Sierpinski-like hierarchical structures [51], [58], the local prey density 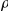 is well-defined.

Acoustic devices allow to measure the density of marine organisms, either locally (e.g., [37]) or over hundreds of kilometers squared instantaneously [60]. Krill density has been observed to fluctuate widely from one location to another and to follow a power-law frequency distribution, of the form 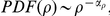 with 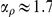 [37]. These large density variations also have a spatial structure that involves many length scales across the landscape [60]. Therefore, to characterize the prey field as a patchwork of regions with different densities, one must specify the sizes of these regions. These length scales (*R* below) represent another important ingredient of the model, as the local density alone is not a space variable. For albatrosses, fairly localized high productivity marine areas occur interspersed with vast oceanic areas of low productivity [10], [54]. We construct a model that captures these properties. In the model, high density regions are numerous but small, and represent overall a small fraction of the total area, corresponding to the tail of the density PDF. On the contrary, a significant area fraction is occupied by a few large regions of very low local density. The density is a continuous variable bounded by a minimal and a maximal value.

The definition of a patch tends to be rather inclusive. We define here a patch as a region of space of uniform prey density, with no limitation on its size and density [64], [65]. Consider a random assembly of non-overlapping, roughly circular patches of varying diameters *R* that are drawn from a frequency distribution 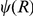 (Figure 3, right). Inside a patch of size *R*, an average number of 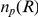 prey are distributed randomly and uniformly. Therefore, the density in a patch is proportional to 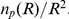 To obtain a medium with fractal properties up to a scale 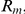 one first distributes *R* according to a truncated power-law distribution:.
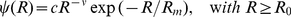
(1)where 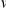 is an exponent related to the fractal dimension, 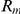 the large cut-off length of the fractal mentioned earlier, and *c* the normalization constant. In addition, *R* is always larger than some length 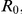 which is the minimum size of a patch 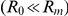 and can be taken as the resolution size. We next assume an algebraic relationship between the size of a patch and the number of prey it contains:
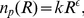
(2)with *k* a constant and  an exponent 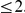 The case 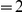 corresponds to a uniform Poissonian medium, where all regions have the same density. As further shown, the albatross data is best fitted by landscapes with negative values of *ε*: large patches have fewer prey. On length scales 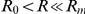, the patch distribution (1) is scale-free, whereas practically no patch has a size much larger than 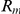. The box-counting method shows that for some parameters 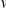 and 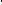 the prey distribution of this model forms a fractal set with dimension 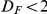 on scales smaller than 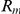 (see Information S1). Restricting ourselves to the case 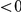 of interest here, one finds that the fractal dimension is given by:
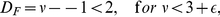
(3)whereas 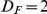 for 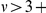. When 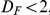 the PDF of the local prey density is an inverse power-law (over a wide range of densities provided that 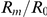 is sufficiently large), with exponent given by:
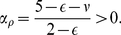
(4)


In this medium, we consider the case of a ballistic predator with constant velocity 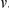 Ballistic motion is the simplest movement behavior and can accurately represent albatross relocations at certain scales [31], [54]. If we assume that there are no correlations between the sizes of neighboring patches, the problem can be simplified to that of a forager flying through a one-dimensional succession of patches (Figure 3, right). The process is easy to simulate numerically: during an elementary time step 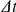
( = 10s, as in [45]), the forager located in a patch of size 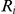 travels a distance 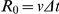 and has therefore a Poissonian probability 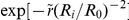 of not finding any prey, with 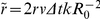 the dimensionless detection radius and 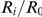 the dimensionless patch size. The process is iterated until the end of a patch is reached, when a new 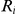 (and therefore a new prey density) is drawn from Equation 1.

After a prey is detected, the forager can either (i) follow its way (“non-responsive search”) or (ii) stay within the same patch for 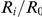 other elementary time steps (“responsive search”). The latter rule mimics area restricted search [30], [33], a behavior that has been observed in wandering albatrosses [31], [54]. With rule (ii), the forager tends to exploit more intensively higher density regions, where detections are more probable.

### Results of the LD and FLD Models

**Figure 4:**
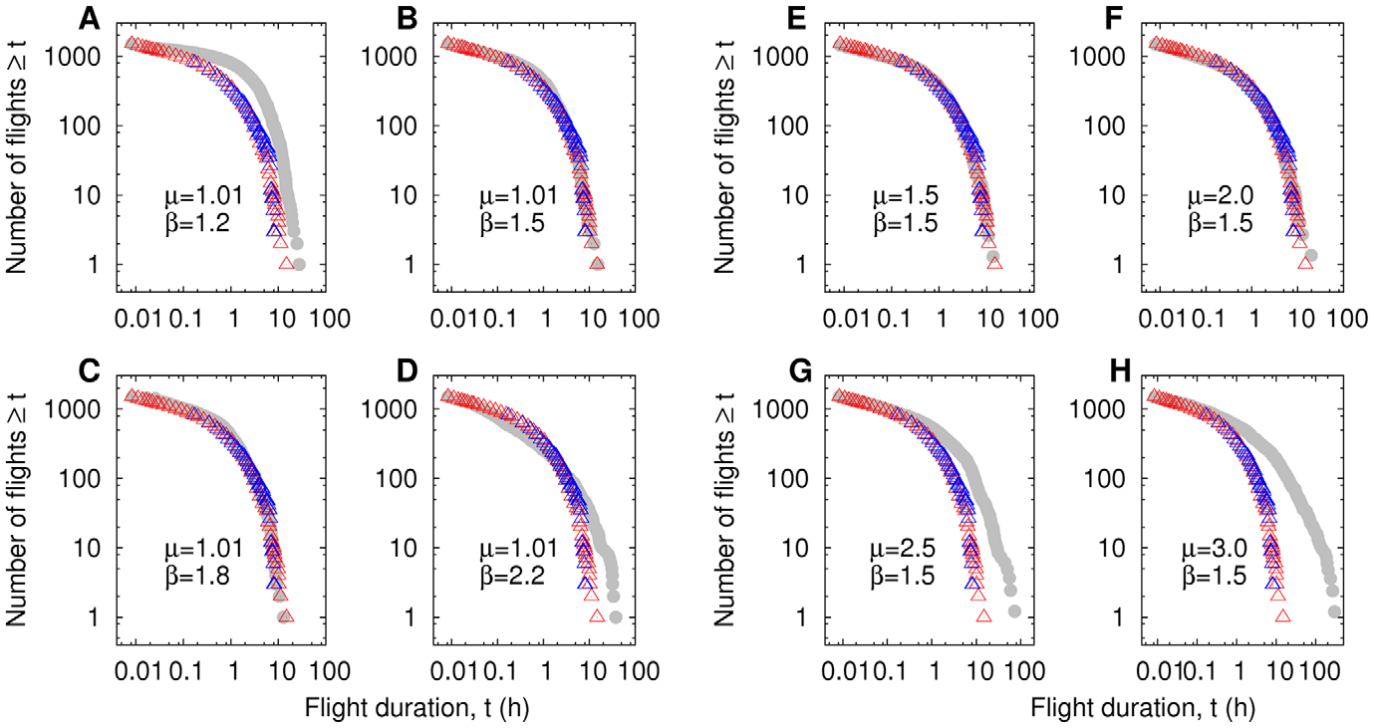
Detections in LD media. (A)-(D): Accumulated histograms of prey detection times (grey circles) for a ballistic model predator (*µ* = 1.01) with *r* = 0.0003 foraging in LD environments (*N* = 5000) of varying fractal dimension at lower scales. Foraging is destructive in all cases. Bird Island data: red triangles, Crozet Islands: blue triangles. Recall that 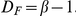 (A) 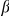 = 1.2 (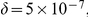 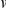 = 0.55), p-value of K-S test on Bird Island: 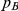 = 0.0045, Crozet Islands: 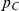 = 4.9e-08; (B) 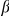 = 1.5 (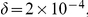 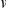 = 0.50), 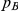 =  0.997, 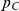 = 0.248; (C) 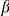 = 1.8 (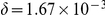, 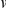 = 0.25), 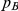 =  0.997, 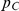 =  0.367 and (D) 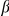 = 2.2 (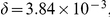 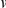 = 0.20), 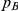 =  0.033, 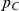 =  0.033. (E)-(H): Same quantities for LD media with fixed 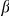 = 1.5 (*N* = 5000, 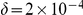) and a model forager following processes with different step length distributions: (E) *µ* = 1.5 (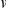 = 0.5), 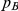 =  1, 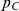 =  0.248; (F) *µ* = 2.0 (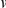 = 0.67), 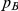 =  0.999, 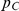 =  0.0995; (G) *µ* = 2.5 (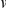 = 0.67), 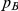 =  0.000955, 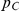 = 4.03e-09 and (H) *µ* = 3.0 (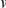 = 0.67), 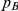 =  6.38e-05, 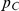 =  1.72e-13.

A ballistic walker foraging through a LD with 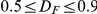 (corresponding to 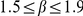) produces a flight duration distribution that fits very well the Bird Island [45] and Crozet Islands [10] albatross data over the entire range (Figure 4A-D). Somewhat surprisingly, no fine tuning of the fractal dimension is needed, as a range of small values of 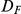 describes the data equally well. In contrast, Lévy dust landscapes with 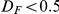 or 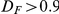 do not produce a good agreement with empirical data. In a given landscape, detection patterns are also robust to changes in the assumptions regarding predator movements. If predators, instead of being ballistic (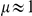), choose step lengths with 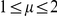, for instance 1.5 and 2, 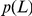 in Figure 4E-H still fits the data very well (LD with 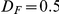). Poor agreement is obtained for 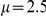 and larger, therefore, albatross data cannot be explained by a Gaussian random walker detecting prey in a fractal media. While a LD fractal prey field gives detection statistics that are qualitatively in excellent agreement with the observed albatross data, estimations of 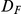 for oceanic prey fields are indeed in the range 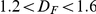 [50], [57], [59]. This quantitative difference prompts us to analyze the FLD model where good agreement can be obtained with 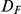 in this range of values.

**Figure 5:**
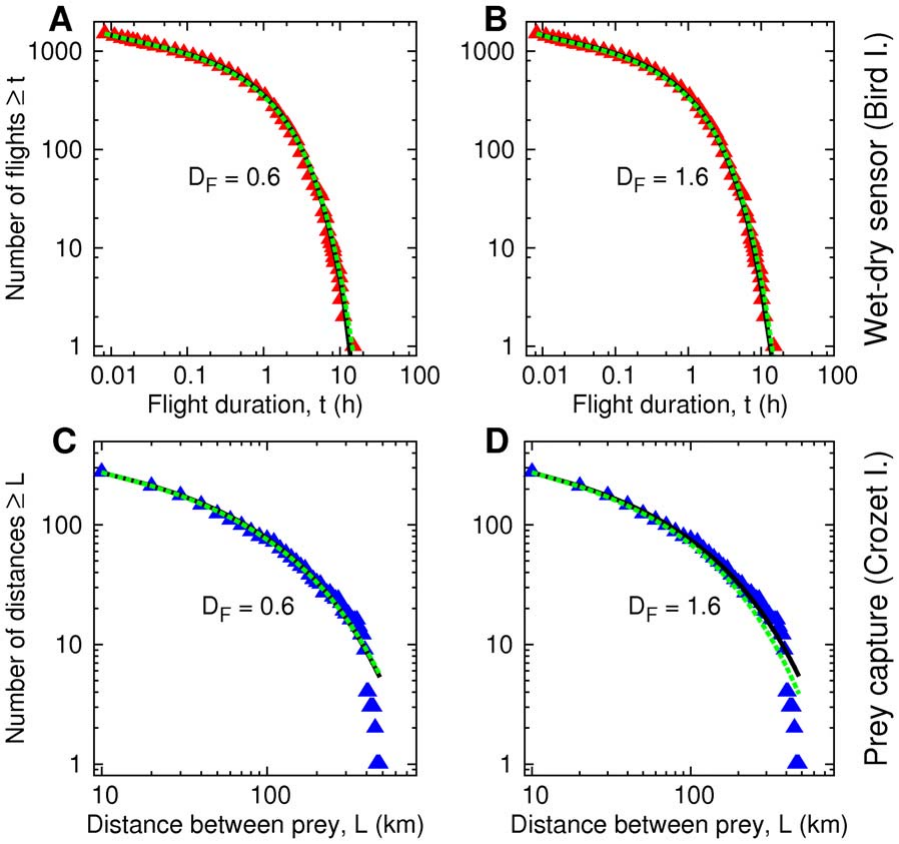
Cumulative distribution of prey detection times/distances obtained by fitting the FLD model to the albatross data (triangles), for two fixed fractal dimensions of the medium (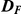 = 0.6 and 1.6). Solid black line: responsive search; green dotted line: non-responsive search. Each curve is plotted with the MLE of the parameters, see Table 1. (A)-(B): Bird Island. (C)-(D): Crozet Islands. The best estimates of the patch size distribution parameters vary little in the different cases: 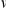 = 1.20 ± 0.05 and 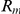 in the range of 160–240 km, independently of 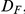 for the whole range considered. A more efficient strategy yields a lower dimensionless detection radius 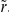

The FLD model gives similar results (Figure 5). First, a range of values of the fractal dimension can fit the data. Secondly, the different foraging behaviors considered can fit the data, too. Table 1 displays, for various values of 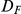 and forager behaviors, the maximum likelihood estimates (MLE) of the exponent 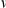 of the patch size distribution, of the cut-off length 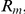 and of the dimensionless detection radius 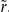 The responsive search scenario describes the data as well as the simple ballistic one. The main difference between the two cases is the value of 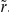 The responsive case is much more efficient since the same prey detection patterns are obtained by a forager with detection radius 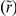 2
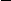
20 times smaller compared with a non-responsive forager in the same medium.

**Table 1:** Maximum likelihood estimates of the Fractal Local Density model parameters (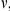 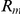 and 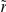) in several scenarii, for each albatross data set.

		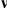	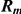 (km)	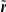	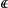	(*P*, *G*)
Bird Island [45]	(wet-dry sensor data)					
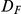 = 0.6	responsive search	1.2	160	1.8	−0.4	(0.69, 43.0)
"	non-responsive search	1.15	180	9.8	−0.45	(0.56, 46.0)
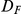 = 1.6	responsive search	1.2	160	5.4	−1.40	(0.51, 47.3)
"	non-responsive search	1.15	180	180	−1.45	(0.58, 45.6)

Note: in the Bird I. case, 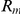 was obtained by converting flight durations into distances assuming 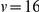
m/s [52], [55].

Importantly, within each data set the MLE of the patch size distribution parameters (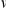 and 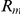) are nearly independent of 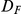 and the foraging scenario. The parameter values found are also strikingly similar across the two albatross data sets. Using an estimate of albatrosses’ speed, 16 m/s [52], [55], the Bird Island flight durations were converted into km. The values of 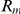 in Table 1 are on the order of hundreds of kilometers, the same order of magnitude as the self-similarity range found in marine landscapes [50], [58]–[60]. Even by assuming that the Bird Island data are accurate for flights longer than 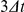 = 30s [45] or 480 m, these values indicate that the albatross prey field is fractal over nearly three logarithmic decades.

In summary, our fractal landscape model produces non-exponential detection patterns and can explain wandering albatross data with realistic parameters. A non-trivial result is that the shape of the flight durations PDF is primarily determined by the patch size distribution 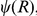 rather than by the fractal dimension 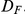 Similarly to the robustness observed in the LD model, the shape of 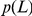 in the FLD model is not altered by modifications in the forager movement strategy.

## Discussion

The foregoing results show the importance of considering predator displacements and prey detection events in unpredictable environments as two different aspects of the same foraging process. We emphasize that detection patterns alone are in general unlikely to inform movement patterns and search strategies. Detection statistics of long-ranging foraging animals in the ocean can be regarded as depending on the size of the regions with uniform density, i.e. a higher level of landscape organization, and not on all the details of the prey field. This result resonates with the current view that marine animals can track meso and submeso-scale seascape features [66]. Our study suggests that detection statistics in both uniform and scale invariant landscapes depend little on the hypothesized predator movement rules, therefore forager search strategies cannot be inferred from detection patterns only.

Wandering albatrosses adjust their movement to cope with overdispersed prey and environmental features at different scales [10], [54]. The two data sets analyzed here can be consistently explained by different foraging models assuming that landings and prey capture are related to prey detection and that prey is fractally distributed from about 200–400 m up to scales of 150−250 km. These scales are in agreement with observations of the distributions of pelagic fish, plankton and squid in the ocean [50], [58], [60]. We infer that albatross prey distribution can be pictured as a random, self-similar assembly of regions with varying sizes and densities (FLD model). The empirical PDF of flight lengths is well reproduced if the size of the aforementioned regions follows a power-law distribution with exponent close to unity (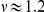, see Table 1). The truncation of very long flights (>200 km) is unavoidable as the prey field tends to be space filling beyond these scales. Our results on landing/take-off activity are consistent with direct prey capture data of wandering albatrosses, suggesting that both are closely related, although not strictly equivalent.

The probability distribution function of the local density of krill, the prey of several top marine predators, is described by an inverse power-law, 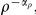 with 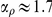 over four decades [37]. It is likely that many other types of organisms, in particular large fish, follow a similar pattern [60]. In the FLD model, along with 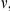 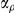 is an important exponent characterizing the prey field. In the two examples of Figure 5-Table 1, where 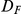 is fixed to 0.6 and 1.6, respectively, we obtain 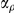 = 1.75 and 1.53 from relation (4). These values are comparable to the empirical exponent 1.7. These results also imply considerable relative variations in albatross prey density, at least of the order of 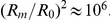


Large fluctuations in prey density have been identified as a possible cause of non-exponentially distributed detections [67]. The FLD model shows that it is indeed the case, if the local density fluctuations are structured in widely different characteristic sizes across many scales. As an example, in the ocean, high productivity areas are separated by larger areas of lower productivity [10], [54]. A simple analytical calculation can show that a forager crossing an heterogeneous medium composed of patches of equal and small sizes, although with power-law distributed prey densities, has an exponential 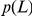 [68]. Hence, not only prey density distributions but the spatial arrangement of prey density fluctuations seem to be a crucial element to obtain non-exponentially distributed detections. In a different context, the study of a model of ballistic particles propagating through Sierpinski-like fractals showed that detection patterns were not exponentials [51]. Our modeled landscapes differ from these Sierpinski gaskets, though, as the fractals considered here are not characterized by a single length scale between nearest prey, an important assumption made in [51]. As noted earlier, no general relation has to be expected between the fractal dimension and detection statistics, which also depend on the kind of fractal structure considered.

Our results also show that random but uniform prey fields should lead to exponential detection patterns. We have identified exponential distributions of flight durations between dives in the thick-billed murre, an Arctic seabird that, unlike the much bigger wandering albatross, forages at small spatiotemporal scales by restricting its search over reduced areas where prey predictability is higher [46]. These observations can be interpreted within our modeling framework: a forager with a high degree of site fidelity performing a search restricted to areas where prey encounter is high should not experience large variations in prey density. Therefore, the detection patterns should come closer to an exponential form than for a species searching over vast oceanic surfaces.

We conclude that detection statistics, as well as other behavioral traits of seabirds [15], can give valuable information on the prey field spatial distributions. Namely, in our examples the frequency distribution of detection times or distances follow a scaling law, 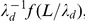 where 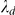 is a typical length between prey detections and depends both on predator movements and the prey field, whereas 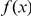 depends on the prey field only. The function 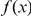 is typically an exponential for uniform prey fields and may involve power-law terms for fractal media. These findings could be useful for disentangling the renewed debate on how organism-environment interactions build up statistical patterns of movement [29], [36], [39], [40] not only in seabirds but in other animals as well.

## Supporting Information

Information S1 **Landscape properties of the Fractal Local Density Model.**
(PDF)
