# Supplementary material for: The Effects of Spatially Heterogeneous Prey Distributions on Detection Patterns in Foraging Seabirds
Source: PLoS One. 2012 Apr 13;7(4):e34317. doi: 10.1371/journal.pone.0034317 (PMC3326003; doi:10.1371/journal.pone.0034317)
Supplement: Information S1 — Landscape properties of the Fractal Local Density Model. (PDF) [file pone.0034317.s001.pdf]

Supporting Information S1 for "The effects of spatially heterogeneous prey distributions on detection patterns in foraging seabirds"

O. Miramontes, D. Boyer and F. Bartumeus

1 Landscape properties of the Fractal Local Density Model

1.1 Calculation of the fractal dimension

In this model, prey are distributed on a plane that is randomly subdivided into non-overlapping patches of heterogeneous diameters (see Materials and Methods, main article). The patch diameter probability distribution function (PDF), $\psi(R)$, is given by the power-law

$$\psi(R) = (\nu - 1)R_0^{\nu-1}R^{-\nu}, \quad R \geq R_0, \quad \nu > 1, \tag{1}$$

and $\psi(R) = 0$ for $R < R_0$, where R_0 is the minimum patch diameter. ($\int_0^\infty \psi(R)dR = 1$.) A patch of size R contains in average $n_p(R)$ prey uniformly and randomly distributed inside the patch. We assume $n_p(R) = kR^\epsilon$ with k a constant and ϵ a scaling exponent. Let $N_0(> 1)$ be the average number of prey that a patch of smallest size R_0 contains. Thus,

$$k = \frac{N_0}{R_0^\epsilon}. \tag{2}$$

If $\epsilon = 2$, the number of prey per unit area, $\rho(R) = n_p(R)/R^2 = kR^{\epsilon-2}$, is independent of R and the medium is uniform. We show below that the prey system can be fractal if $\epsilon < 2$, when $\rho(R) \to 0$ for large patches, and calculate its fractal dimension D_F using the standard box-counting method [1]. For a fractal medium, we expect a relation of the form

$$\mathcal{N}(\sigma) \sim \sigma^{-D_F}, \tag{3}$$

where $\mathcal{N}(\sigma)$ is the average number (per patch) of non-overlapping square boxes of length σ that cover all the prey ($\sigma > R_0$ in the following). Two cases are distinguished below: $0 \leq \epsilon < 2$ (big patches have more prey) and $\epsilon < 0$ (big patches have fewer prey). Although fits to the albatross data suggest that the prey system has $\epsilon < 0$ (see main article), we also present the results of the other case for completeness.

a) Case $0 \leq \epsilon < 2$.

If $\epsilon > 0$, there are always more than one prey per patch. As the prey density $\rho(R) = kR^{\epsilon-2}$ is uniform inside the patch, the typical distance between neighboring prey is $d(R) = 1/\rho(R)^{1/2}$, which is $< R$. Consider square boxes of size σ, larger than the smallest patch diameter R_0. If $d(R)$ does not exceeds σ, the patch can be completely covered by $(R/\sigma)^2$ boxes on average [this number is < 1 if $R < \sigma$, meaning that one box covers more than one patch on average]. If on the other hand $d(R) > \sigma$, $n_p(R) = kR^\epsilon$ boxes are needed in average to cover all the prey inside the patch. For a fixed σ, there is a patch size R^* such that $d(R^*) = \sigma$. Using notation (2),

$$R^* = R_0 \; N_0^{1/(2-\epsilon)} (\sigma/R_0)^{2/(2-\epsilon)}. \tag{4}$$

From the considerations above, one can write

$$\begin{aligned} \mathcal{N}(\sigma) &= \int_{R_0}^{R^*} dR\psi(R) \left(\frac{R}{\sigma}\right)^2 + \int_{R^*}^{\infty} dR\psi(R) kR^\epsilon \\ &\equiv \mathcal{N}_1(\sigma) \; + \; \mathcal{N}_2(\sigma). \end{aligned} \tag{5}$$

Several cases are to be distinguished.

If the patch exponent ν is > 3, one finds

$$\mathcal{N}_1(\sigma) \simeq \frac{\nu-1}{\nu-3} \left(\frac{\sigma}{R_0}\right)^{-2}, \quad \sigma \gg R_0 \tag{6}$$

$$\mathcal{N}_2(\sigma) = \frac{\nu-1}{\nu-\epsilon-1} N_0^{\frac{3-\nu}{2-\epsilon}} \left(\frac{\sigma}{R_0}\right)^{-2\frac{\nu-\epsilon-1}{2-\epsilon}}. \tag{7}$$

Since $N_0 > 1$ and $2(\nu - \epsilon - 1)(2 - \epsilon) > 2$, then $\mathcal{N}_1 \gg \mathcal{N}_2$ when $\sigma \gg R_0$. Comparing with the definition (3), one concludes:

$$D_F = 2. \tag{8}$$

Hence, if $\nu > 3$, the prey system is bidimensional, although with a non-uniform density.

If $\epsilon + 1 < \nu < 3$, one finds, for $\sigma \gg R_0$:

$$\mathcal{N}(\sigma) = \left[\frac{1}{3-\nu} + \frac{1}{\nu-\epsilon-1}\right] (\nu-1) N_0^{\frac{3-\nu}{2-\epsilon}} \left(\frac{\sigma}{R_0}\right)^{-2\frac{\nu-\epsilon-1}{2-\epsilon}}. \tag{9}$$

Therefore,

$$D_F = 2\frac{\nu-\epsilon-1}{2-\epsilon}, \tag{10}$$

which is lower than 2.

If $\nu < 1 + \epsilon$, the average prey number per patch is infinite and the medium can not be described as a fractal. This case will not be considered here.

b) Case $\epsilon < 0$.

The calculation above must be modified for negative values of ϵ. The average prey number is < 1 in patches larger than

$$R_c = R_0 N_0^{-1/\epsilon}. \tag{11}$$

One can figure these low density patches as containing one prey with probability kR^ϵ and zero prey with probability $1 - kR^\epsilon$. Hence, if $R > R_c$, $d(R)$ does not represent the distance between neighboring prey as in the previous case. Let us restrict our analysis to boxes of size $\sigma > R_c$ in the following. A patch with $R < R_c$ is completely covered (and therefore all its prey) by $(R/\sigma)^2(< 1)$ boxes. A patch with $R_c < R < \sigma$ is covered by $(R/\sigma)^2$ boxes, too, and has a probability $kR^\epsilon(< 1)$ of containing one prey. A patch with $R > \sigma$ has a probability kR^ϵ of containing one prey and therefore requires kR^ϵ boxes in average to be covered. Therefore:

$$\begin{aligned} \mathcal{N}(\sigma) &= \int_{R_0}^{R_c} dR\ \psi(R) \left(\frac{R}{\sigma}\right)^2 \\ &\quad + \int_{R_c}^{\sigma} dR\ \psi(R) \left(\frac{R}{\sigma}\right)^2 kN^\epsilon \\ &\quad + \int_{\sigma}^{\infty} dR\ \psi(R)\ kR^\epsilon \\ &\equiv \mathcal{N}_1(\sigma)\ +\ \mathcal{N}_2(\sigma) +\ \mathcal{N}_3(\sigma). \end{aligned} \tag{12}$$

Performing the integrals, one finds ($\sigma \gg R_0$):

$$\mathcal{N}_1(\sigma) \simeq \begin{cases} \frac{\nu-1}{\nu-3} \left(\frac{\sigma}{R_0}\right)^{-2}, & \nu > 3 \\ \frac{\nu-1}{3-\nu}\ N_0^{(\nu-3)/\epsilon} \left(\frac{\sigma}{R_0}\right)^{-2}, & \nu < 3, \end{cases} \tag{13}$$

$$\mathcal{N}_2(\sigma) \simeq \begin{cases} \frac{\nu-1}{\nu-3-\epsilon}\ N_0^{(\nu-3)/\epsilon} \left(\frac{\sigma}{R_0}\right)^{-2}, & \nu > 3+\epsilon \\ \frac{\nu-1}{3+\epsilon-\nu}\ N_0 \left(\frac{\sigma}{R_0}\right)^{-(\nu-\epsilon-1)}, & \nu < 3+\epsilon, \end{cases} \tag{14}$$

$$\mathcal{N}_3(\sigma) = \frac{\nu-1}{\nu-\epsilon-1}\ N_0 \left(\frac{\sigma}{R_0}\right)^{-(\nu-\epsilon-1)}, \quad \forall\ \nu > 1. \tag{15}$$

For a fixed patch exponent ν, the fractal dimension is given by the leading term(s) in eqs.(13)-(15), *i.e.*, the term(s) with the slowest power-law decay as a function of σ/R_0. One obtains:

$$D_F = 2 \qquad \text{for } \nu > 3+\epsilon \tag{16}$$

$$D_F = \nu - \epsilon - 1 < 2 \qquad \text{for } \nu < 3+\epsilon. \tag{17}$$

1.2 Distribution of the local density

In this subsection, we show that in the Fractal Patch model with patch size distribution of the form $\psi(R) \sim R^{-\nu}$ and $\epsilon < 2$, the probability distribution

function $f(\rho)$ of the local density ρ is also a power-law, $f(\rho) \sim \rho^{-\alpha_\rho}$, with exponent:

$$\alpha_\rho = \frac{5-\epsilon-\nu}{2-\epsilon}. \tag{18}$$

(See equation (4), main article). A motivation for calculating α_ρ is that recent acoustic measurements have shown that the prey of some top marine predators have densities that are power-law distributed in space [2].

The probability that a small region of the plane has a density larger than a value ρ is equal to the fraction area occupied by patches smaller than R, with $\rho = kR^{\epsilon-2}$. Namely,

$$\int_\rho^{\rho_0} f(x)dx = \int_{R_0}^{R} x^2\psi(x)dx, \tag{19}$$

with $\rho_0 = \rho(R_0)$ the largest density, that is found in the smallest patches. Taking the derivative of eq.(19) with respect to ρ, one obtains

$$f(\rho) \sim \left|\frac{dR}{d\rho}\right| \; R^{2-\nu}. \tag{20}$$

Using the relation $R \sim \rho^{1/(\epsilon-2)}$ yields to the above mentioned result.

If $\nu < 3$, then $\alpha_\rho > 1$ from eq.(18): The presence of very large patches of low densities produce a sharp increase of $f(\rho)$ as $\rho \to 0$. This is akin to the situation encountered in [2], where $\alpha_\rho \approx 1.7$ was observed for krill densities. A realistic description of prey in the sea requires that the patch size distribution $\psi(R)$ is exponentially cut-off at large scales, with few patch exceeding a value R_m (see eq.(1), main article). This puts a lower bound on ρ and ensures that $f(\rho)$ is integrable in the small ρ region.

If $\nu > 3$, then $\alpha_\rho < 1$. The medium is more homogeneous than in the previous case due to the relative scarcity of large patches. Even if the patch size distribution has no large size cut-off (*i.e.* $\psi(R)$ is a pure power-law), $f(\rho)$ remains integrable in $\rho = 0$. At large densities, on the other hand, the distribution $f(\rho)$ is very "flat". It drops to zero, however, once the highest density in the system, ρ_0, is reached.

References

[1] B. B. Mandelbrot. The Fractal Geometry of Nature. W. H. Freeman (1983).

[2] D. W. Sims *et al.* Scaling laws of marine predator search behaviour. *Nature* **451**, 1098–1102 (2008).
